# Biphasic decay kinetics suggest progressive slowing in turnover of latently HIV-1 infected cells during antiretroviral therapy

**DOI:** 10.1186/1742-4690-5-107

**Published:** 2008-11-26

**Authors:** Marek Fischer, Beda Joos, Barbara Niederöst, Philipp Kaiser, Roland Hafner, Viktor von Wyl, Martina Ackermann, Rainer Weber, Huldrych F Günthard

**Affiliations:** 1 University Hospital Zürich, Division of Infectious Diseases, Rämistrasse 100, 8092 Zürich, Switzerland

## Abstract

**Background:**

Mathematical models based on kinetics of HIV-1 plasma viremia after initiation of combination antiretroviral therapy (cART) inferred HIV-infected cells to decay exponentially with constant rates correlated to their strength of virus production. To further define in vivo decay kinetics of HIV-1 infected cells experimentally, we assessed infected cell-classes of distinct viral transcriptional activity in peripheral blood mononuclear cells (PBMC) of five patients during 1 year after initiation of cART

**Results:**

In a novel analytical approach patient-matched PCR for unspliced and multiply spliced viral RNAs was combined with limiting dilution analysis at the single cell level. This revealed that HIV-RNA^+ ^PBMC can be stratified into four distinct viral transcriptional classes. Two overlapping cell-classes of high viral transcriptional activity, suggestive of a virion producing phenotype, rapidly declined to undetectable levels. Two cell classes expressing HIV-RNA at low and intermediate levels, presumably insufficient for virus production and occurring at frequencies exceeding those of productively infected cells matched definitions of HIV-latency. These cells persisted during cART. Nevertheless, during the first four weeks of therapy their kinetics resembled that of productively infected cells.

**Conclusion:**

We have observed biphasic decays of latently HIV-infected cells of low and intermediate viral transcriptional activity with marked decreases in cell numbers shortly after initiation of therapy and complete persistence in later phases. A similar decay pattern was shared by cells with greatly enhanced viral transcriptional activity which showed a certain grade of levelling off before their disappearance. Thus it is conceivable that turnover/decay rates of HIV-infected PBMC may be intrinsically variable. In particular they might be accelerated by HIV-induced activation and reactivation of the viral life cycle and slowed down by the disappearance of such feedback-loops after initiation of cART.

## Background

Current combination antiretroviral therapy (cART) does not attack virus-infected cells themselves but targets viral replication at major steps in the viral life cycle [1]. Thus, the decline of HIV-1 plasma viremia induced by cART has been interpreted to reflect cell-specific decay rates of HIV-infected cells with different life-spans and rates of virus production [2,3]: A first phase of decay, perceptible within the first weeks of cART, has been attributed to the initial loss of productively infected activated T-lymphocytes. Due to their intrinsically short life-span [4] and to direct viral and immunity-mediated cytopathic effects [5], these cells are prone for rapid cell-death.

Later phases of decay were thought to reflect expanded life-spans of virus producing macrophages or memory T-lymphocytes [5]. In addition, latently infected cells reactivated to productivity, may also contribute to the pool of HIV-virions observed in later decay phases [2,3]. When viremia levels fall below the threshold of detection, persisting infection is primarily due to a long lived reservoir of latently infected CD4^+ ^cells [6-8].

Mathematical models based on plasma viremia only indirectly allow inferring kinetics of latently infected cells which lack virus production. Direct quantification of latently infected cells ex vivo has commonly been attained by viral outgrowth assays of resting CD4^+^-T-lymphoctyes [6]. These bioassays relying on inducibility and longevity of donor and indicator cells may underestimate numbers of latently infected cells. Accordingly, their frequencies during cART have been estimated to be very low, in the order of 1 in 10^6 ^lymphocytes [8]. Further characterization of the cells constituting the latent reservoirs has revealed that only a very low percentage of resting CD4 T-cells carrying HIV-DNA can be induced ex vivo to give rise to viral transcription[9] or antigen production [10].

This contrasts with comparatively high levels of cell-associated viral RNA (hundreds to thousands of viral RNA copies/10^6 ^cells) observed in peripheral blood of patients on cART, even in the absence of detectable plasma viremia [11-14]. Recently, evidence has accumulated that HIV-RNA persisting during cART may to a large extent reflect basal transcription in latently infected cells devoid of virion production [9,12,15-17]. Such bulk measurements of cellular HIV-1 RNAs, despite their potential to monitor viral activity far beyond undetectable viremia [15], have considerable shortcomings, namely their lack of unambiguous differentiation between viral transcription in latently versus productively infected cells.

In the present study we refined the analysis of HIV-transcription, by combining highly sensitive PCR assays for a panel of unspliced (UsRNA) and multiply spliced (MsRNA) HIV-RNA species with limiting dilution end-point analysis of PBMC. Using this approach, we were able to dissect the population of HIV-RNA^+ ^PBMC according to their level of viral transcription and to determine frequencies and kinetics of cells expressing proviral DNA at different rates.

## Results

### Analysis of HIV-1 transcription in serial dilutions of PBMC

Individually adjusted RT-PCR targeting HIV-1 nucleic acids was performed on serial dilutions of PBMC assessing HIV-DNA, UsRNA, total MsRNA and MsRNA-tatrev or MsRNA-nef [15]. In parallel to testing total RNA extracts, vRNA-ex representing cell-associated viral particles, was quantified in separate replicate specimens [12,18]. Limiting dilution analysis of HIV-RNA^+ ^cells was performed to compute their frequencies which also allowed determining the average per-cell expression of HIV-RNA.

**Figure 1:**
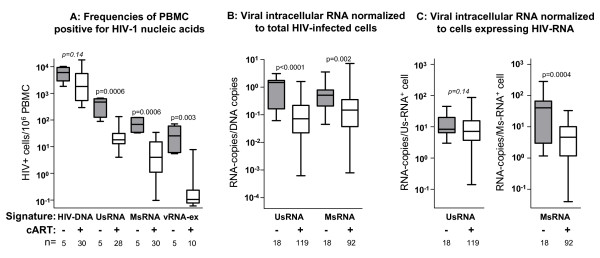
**Antiretroviral therapy mediated decreases in HIV-infected cells and average cellular viral transcriptional activity**. HIV-RNA (UsRNA, MsRNA, vRNA-ex), HIV-DNA levels and frequencies of PBMC positive for HIV-RNAs were measured before start of cART (grey boxes) and at six time-points during treatment (white boxes). Signature signifies the type of viral nucleic acid measured for determination of infected cell-numbers. Sample sizes in each group (n = sample numbers, analysis of 5 patients, one time point before cART, six time-points during therapy, only data of time points with PCR-positive samples were included) are indicated below diagrams and p-values of Mann-Whitney comparison of treated versus untreated groups are indicated above. Groups are displayed as "box and whiskers" showing the median, 75% percentiles and range of each data set. A: Frequencies of total infected PBMC, as represented by HIV-DNA levels and frequencies of PBMC expressing viral RNAs determined by limiting dilution as described in figure 2. (B: Average per-cell expression of intracellular viral RNAs (UsRNA, MsRNA) normalized to HIV-DNA (representing the total number of HIV-infected cells). C: Average per-cell expression of intracellular viral RNAs normalized to the numbers of PBMC expressing viral RNA. To favour sampling of balanced average populations, solely viral RNA measurements from specimens containing more than 10^6 ^PBMC were analyzed in B and C (n = 2–6 per time-point and patient).

As shown in figure 1A, the numbers of cells expressing UsRNA or MsRNA experienced significant decreases (p = 0.0006) as a result of antiretroviral treatment while decrease of total HIV-infected PBMC was less pronounced (p = 0.14). Paired analysis throughout the course of observation (one-way Anova-Friedman test, comparison of frequencies of HIV-DNA^+^, UsRNA^+^, MsRNA^+^, vRNA^+ ^cells per patient and per time-point; p < 0.0001) showed that total HIV-infected PBMC exceeded cells expressing viral RNA, which revealed a preponderance of transcriptionally silent provirus in peripheral blood. Moreover, cells expressing UsRNA were invariably more frequent than cells expressing MsRNA and the latter were more frequent than cells positive for UsRNA-ex. These findings provide evidence for the existence of cells expressing solely UsRNA and cells expressing MsRNA and presumably also UsRNA and a third very rare population of cells positive for vRNA-ex.

To further characterize HIV-RNA expression, average intracellular per-cell expression of UsRNA and MsRNA was calculated by normalizing RNA copy numbers to frequencies of total HIV-infected PBMC (figure 1B) or to cells actually expressing viral RNA (figure 1C). Using either mode of calculation, per-cell expression of MsRNA was significantly lower in samples obtained during cART as compared to samples from untreated patients (total cells: 4-fold decrease; p = 0.002; figure 1B, HIV-RNA^+ ^cells 9-fold decrease; p = 0.0004; figure 1C). Reduction of per-cell UsRNA-expression during treatment attained high statistical significance when normalized to total HIV-infected PBMC (20-fold; p < 0.0001; figure 1B) but was perceptible only as a trend when UsRNA-expression was normalized to UsRNA^+ ^cells (1.2-fold, p = 0.14; figure 1C). Thus, per-cell MsRNA expression and to a lesser extent also UsRNA-expression appeared to split up into two discernible states. From these findings the following implications can be inferred:

i) Several classes of HIV-infected cells differing in their viral transcriptional activity co-occur before therapy. After initiation of cART cells with lower RNA content appear to outlast cells expressing higher levels of viral RNA.

ii) Three cell classes can be dissected directly using limiting cell-dilution, by virtue of their hierarchical distribution of frequencies: a class which expresses solely UsRNA, one class expressing MsRNA and presumably also UsRNA and a class of cells positive for vRNA-ex.

iii) Cells expressing MsRNA may host one or more subcategories of infected cells with lower and higher viral transcriptional activity.

### A model for stratification of viral RNA content in HIV-1 infected PBMC

To account for the observed complexity of HIV-RNA expression in PBMC, we designed a simple model to resolve and identify cell categories of different transcriptional states. In particular, the data presented above suggest the coexistence of four main classes of HIV-RNA^+ ^cells namely, I_Low _(low transcriptional activity), II_Medium _(intermediate transcriptional activity), II_High _(high transcriptional activity) and III_Extra_(ongoing extracellular virion shedding).

#### I_Low_

*HIV-1 infected cells containing solely UsRNA*. The existence of this cell class is deduced from our observations that UsRNA-positive cells were invariably more frequent than cells expressing MsRNA.

#### II_Medium_

*HIV-1 infected cells expressing MsRNA at low levels*. Evidence for this class of cells is based on significant differences in per-cell MsRNA content in PBMC from patients on cART as compared to untreated patients. It is highly likely that such cells express UsRNA because MsRNAs are obligatorily derived from primary unspliced HIV-transcripts [19].

#### II_High_

*HIV-1 infected cells with elevated viral transcription. S*ignificantly higher relative expression of both UsRNA and MsRNA in untreated versus treated patients, suggests frequent presence of cells exhibiting Tat/Rev-mediated transcriptional activation [20,21] at baseline.

### III_Extra_

*Cells carrying virion-enclosed HIV-1 RNA*. Such cells to a major extent represent productively infected cells in a state of ongoing or recent burst of viral shedding as previously demonstrated by their association with activated viral transcription [12,15,16].

**Figure 2:**
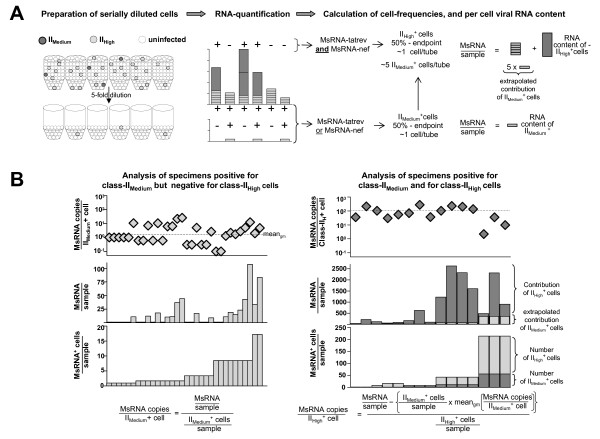
**Outline of experimental strategy. **A: Algorithm for combining limiting dilution of cells with RT-PCR. HIV-RNAs (in this example MsRNAs) of serial 5-fold dilutions of cells (left panel) are measured by RT-PCR (middle panel). Analysis of replicates of each dilution (right panel) reveals both the viral RNA content and the frequencies (estimated by 50% end-points) HIV-RNA^+^ cells. Applying criteria listed in table 1, in this case expression of either MsRNA-tatrev or MsRNA-nef (class-II_Medium_) and expression of both MsRNA-tatrev and MsRNA-nef (class-II_High_), cell classes differing in HIV-RNA content can be discerned. Specific HIV-RNA expression in each class of MsRNA+ cells can then be normalized by dividing MsRNA copies by the numbers of infected cells. In specimens positive for class-II_High_ cells which always contain class-II_Medium_, the contribution of class-II_Medium_ needs to be considered (see formulas in panel B). Note that analysis of UsRNA contents in different cell-classes followed the same schemes. B: Analysis of specific MsRNA per-cell expression exemplified for patient 112. MsRNA expression (middle panels) was normalized to the number of HIV-RNA^+^ cells (bottom panels) resulting in MsRNA expression per cell (top panels). The left three panels comprise specimens positive for class-II_Medium_ expression only. In the right three panels indicating specimens positive for class-IIHigh MsRNA, the average contribution of class-II_Medium_^+^ cells (light grey bars) was subtracted from MsRNA copy numbers before normalization to the number of class-II_High_ + cells. The dotted lines in the top panels show the geometric means (mean_gm_) of all data-points. Note that RNA copies per sample and frequencies of MsRNA^+^ cells (middle and bottom panels) are depicted in a linear scale which may result in column heights hardly discernible from zero. Formulas at the bottom describe the calculations performed. Bars show PCR results of separate replicates of PBMC dilutions, horizontal axes in the diagrams have no dimension

Applying distinct criteria as compiled in table 1, allowed to calculate the number of cells allocated to each cell-class for each specimen containing viral RNA. Thus frequencies of cell-classs were calculated during the course of cART. By using our dataset comprising 476 HIV-RNA^+ ^specimens of total RNA extracts, relative per-cell expression of UsRNA and MsRNA in the three transcriptional categories I_Low_, II_Medium _and II_High _could be calculated as outlined in figure 2.

**Table 1:** Criteria to assign experimental specimens to viral transcriptional classes

			No of unique specimens^A ^for each patient				
Class	Presence of viral RNA	Further criteria	#103	#104	#110	#111	#112
I_Low_	UsRNA^+^	MsRNA	26	46	17	57	34

II_Medium_	MsRNA^+^	Residual (<2 copies/specimen)^B ^content of one type of MsRNA, Ms-tatrev **or **MsRNA-nef	45	51	56	12	32
II_High_	MsRNA-tatrev^+ ^MsRNA-nef^+^	Significant (≥ 2 copies/specimen)^B ^content of two types of MsRNA, MsRNA-tatrev **and **MsRNA-nef	28	33	19	5	15

III_R_	vRNA-ex^+^	Residual (<2 copies/specimen)^B ^content of vRNA-ex	14	1	5	4	5
III_Extra_	vRNA-ex^+^	Significant (≥ 2 copies/specimen)^B ^content of vRNA-ex	25	11	11	5	5

^A ^Unique specimens in an expression class were defined as specimens devoid of cells with higher expression category: Thus unique class-I_Low _positive samples are devoid of class-II_Medium _and class-II_High _cells, unique class II_Medium _specimens are devoid of class-II_High_.

^B ^Distinction between residual and significant viral RNA content was based on the following rationale: At the endpoint of dilution series of infected cells, specimens containing cells with viral RNA content close to one copy per cell will give PCR-positive readout only in single replicates with considerable variations in ct-values. These data were censored to one copy per specimen as described in Methods. Conversely PCR-readouts ≥ 2 copies/specimen reflect measurements where quantification has been found to be reliable in this dataset.

### Distinct transcriptional signatures of HIV-infected PBMC

**Figure 3:**
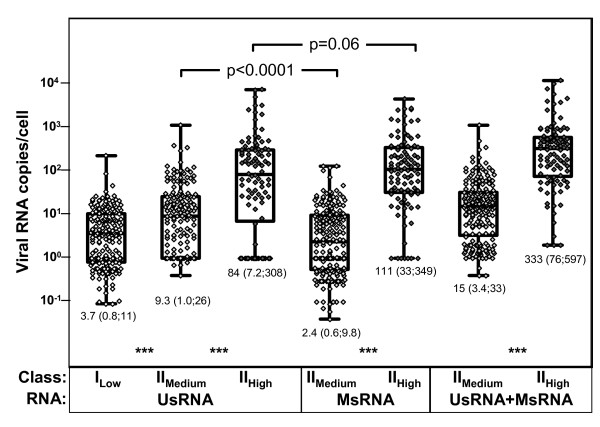
**Transcriptional signatures in HIV-infected PBMC *in vivo***. Per-cell viral RNA expression levels as calculated after assignment of specimens to the transcriptional classes I_Low_, II_Medium _and II_High _(table 1). Only unique specimens devoid of cells of a higher expression category were analyzed in each expression class. Thus unique class-I_Low _positive samples are devoid of class-II_Medium _and class-II_High _cells, unique class II_Medium _specimens are devoid of class-II_High_. Data were pooled from all patients because separate analyses of single patients showed similar distributions (data not shown). "Box and whiskers" show the ranges, medians and interquartile ranges of the data displayed by single symbols. Numbers below columns indicate medians (quartiles), p-values above columns show significance levels of paired (Wilcoxon signed rank test) comparisons. *** indicates p-values < 0.0001 of (unpaired) Mann-Whitney testing between neighbouring columns.

Analysis of viral RNA per-cell contents (figure 3) confirmed that relative expression of HIV-RNA increased in the three transcriptional categories I_Low_, II_Medium _and II_High_. Median viral RNA expression ranged from 3.7 HIV-RNA copies/cell in class-I_Low _to 15 copies/cell in class-II_Medium _and to 333 copies/cell in class-II_High_. Notably, in class-II_Medium _UsRNA expression was approximately four times higher than MsRNA expression (p < 0.0001, Wilcoxon signed rank test), whereas class-II_High _showed an inverse pattern with MsRNA expression equaling or slightly exceeding UsRNA expression (p = 0.06; Wilcoxon signed rank test). Thus class-II_Medium _and class-II_High _displayed different viral transcriptional signatures.

To further validate our stratification of HIV-infected PBMC, relative RNA contents of UsRNA and MsRNA in I_Low_, II_Medium _and II_High _were compared in specimens obtained prior to and during therapy. In the stratum with basal viral transcription of exclusively UsRNA (I_Low_), per-cell viral RNA contents did not differ between baseline and cART (geometric mean, 95%CI, baseline = 2.1, 1.1–3.9 copies/cell; n = 22; on cART = 3.1, 2.4–4.0 copies/cell; n = 158, Mann Whitney test p = 0.22) indicating that this cell-class did not comprise additional subcategories. Similarly, in class II_Medium _cells, per cell content of UsRNA plus MsRNA did not reveal a statistically significant difference when baseline samples were compared to specimens obtained during cART (geometric mean, 95%CI, baseline = 26, 13–47 copies/cell; n = 26, on cART = 14, 11–18 copies/cell; n = 170, Mann Whitney test p = 0.06). Since samples obtained during cART also comprised study week 2, when plasma viremia had not yet stabilized, we also performed comparison of baseline samples to cART without the specimens from week 2. Similarly this analysis did not reveal statistically significant differences between the two groups (data not shown, Mann-Whitney test, p = 0.11). Thus, viral RNA expression in class-II_Medium _cells did not experience evident changes during the course of antiretroviral therapy.

Conversely, viral RNA content was 5× higher (Mann Whitney test p < 0.0001) in class-II_High _at baseline (geometric mean, 95%CI= 532, 334–846 copies/cell; n = 50) as compared to on-therapy samples (geometric mean, 95%CI = 102, 66–158 copies/cell, n = 50).

This shows that categorization of cells expressing MsRNA into the classes II_Medium _and II_High _was still not sufficient to delineate the full scale of viral transcriptional patterns. On a biological level, this finding provides evidence that class-II_High _in untreated patients may harbor a subcategory of HIV-infected cells expressing hundreds of viral RNA copies per cell which likely represents productively infected lymphocytes. Due to limitations in sample size and resolution, transcriptional class-II_High _could not be further dissected. However, we observed that cells expressing significant amounts of vRNA-ex (class-III_Extra_, table 1), a surrogate of productive HIV-infection [12,15,16], occurred primarily before initiation of cART and were in general rarer than class-II_High _cells. Hence it is conceivable that class-III_Extra _represents a productively infected subcategory of class-II_High_. Because the procedure for measuring vRNA-ex necessitates nucleolytic digestion of intracellular RNA and precludes simultaneous quantification of intracellular MsRNA and UsRNA, co-localization of class-III_Extra _cells with class-II_High _in a given sample could not be tested. A minor subcategory of cells harboring vRNA-ex at very low levels (class-III_R_, table 1) was not further characterized because it was likely that these cells may not be HIV-infected but carry passively absorbed plasma virus [12].

### Kinetics of HIV-1 infected PBMC during cART

**Figure 4:**
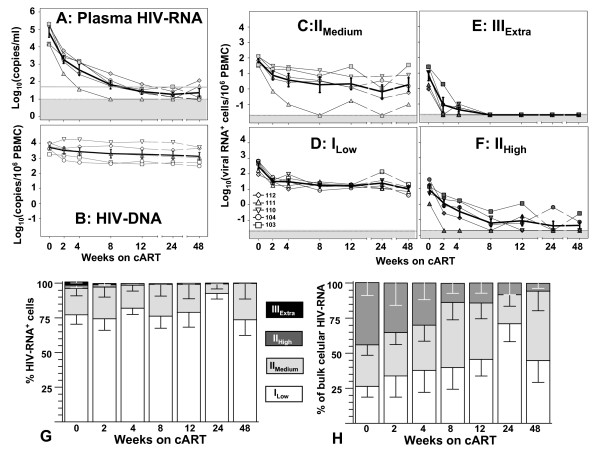
**Kinetics of HIV-1 during cART**. A: Plasma viremia shown by log_10 _transformed plasma RNA copy numbers. B: Total HIV-1 infected cells shown by log_10 _transformed copy numbers of HIV-1 DNA. C; D: Latently infected cells shown by log_10 _transformed numbers of HIV-RNA^+ ^PBMC of cell classes I_Low _(D) and II_Medium_(C). E, F: Cells with increased viral transcription shown by log_10 _transformed numbers of class II_High _(F) and III_Extra _(E). G: Mean (± sem) distribution of HIV-RNA^+ ^cells during cART. H: Mean (± sem) contribution of different HIV-RNA^+ ^cell classes to bulk cellular viral RNA as assessed by the sum of UsRNA and MsRNA in total RNA extracts. Data in A-F are depicted and connected with thin black lines for each patient (see symbols in the bottom of panel D). Dotted lines and the grey shaded bars in panels A and C-F show the estimated detection limit of limiting dilution analyses for HIV-RNA^+ ^PBMC. Symbols coinciding with detection limits signify time points with undetectable viral RNA. Thick black lines depict (log_10 _transformed) means (± sem) of the pooled data from all 5 patients. The grey line in panel A indicates the clinically used threshold of 50 RNA copy per ml.

Turnover and kinetics of HIV-1^+ ^PBMC were analyzed and compared to the decay of plasma viremia as shown in figure 4 and table 2.

**Table 2:** Turnover characteristics of HIV-1 RNA^+ ^PBMC during cART

		Cell numbers^a^		Decaying cell fraction	
Class	Patient	Baseline (N_0_)	Plateau on cART (N_P_)	(1-N_P_/N_0_)	T_1/2 _(days)^b^
I_Low_	103	470	39	91.7%	0.7
	104	661	11	98.3%	3.8
	110	582	29	95.0%	2.5
	111	164	14	91.5%	2.5
	112	89	11	87.6%	6.3
	
	All^C^:	393 ± 114	21 ± 5.6	93 ± 1.8%	3.2 ± 0.9

II_Medium_	103	36	15	58.4%	4.6
	104	125	2.3	98.2%	4.3
	110	122	8.2	93.3%	6.3
	111	33	0.1	99.7%	2.4
	112	68	1.3	98.1%	3.2
	
	All^C^:	76 ± 20	5.4 ± 2.8	90 ± 7.9%	4.1 ± 0.7

II_High_	103	14	≤ 0.01	≥ 99%	8.2
	104	39	≤ 0.01	≥ 99%	2.5
	110	4.9	≤ 0.01	≥ 99%	13
	111	1.1	≤ 0.01	≥ 99%	1.1
	112	18	≤ 0.01	≥ 99%	2.3
	
	All^C^:	15 ± 6.6	≤ 0.01	≥ 99%	5.4 ± 2.3

III_Extra_	103	25	≤ 0.01	≥ 99%	3.8
	104	25	≤ 0.01	≥ 99%	1.7
	110	11	≤ 0.01	≥ 99%	0.7^D^
	111	1.7	≤ 0.01	≥ 99%	0.7^D^
	112	0.9	≤ 0.01	≥ 99%	0.7^D^
	
	All^C^:	13 ± 5.3	≤ 0.01	≥ 99%	1.5 ± 0.6

Total HIV^+ ^(HIV-1 DNA)	103	1808	326	82%	22
	104	4030	370	91%	4.0
	110	8533	2488	71%	174
	111	5965	938	84%	17
	112	10094	4853	52%	1.1
	
	All^C^:	6086 ± 14939	1795 ± 859	76 ± 6.8	44 ± 32

^A^HIV-1^+ ^cells/10^6 ^PBMC

^B^Decay half-lives were calculated assuming one phase exponential decay to a plateau according to the equations; N_t _= (N_0 _- N_P_) × e^-Kt ^+ N_P_; t = time (days), N_0 _= Cell number at baseline; N_P _= Cell number at plateau; N_t _= Cell number at time t; T_1/2 _= LN(0.5)/(-K). Decay constants were constrained between 0 and 1, to exclude decay half-lifes shorter than 0.7 days to account for the limited data points available during periods of initial decay

^C^Mean ± standard error (sem) of data from all five patients.

^D^Exact determination by fitting of class III_Extr _could only be achieved for patients 103 and 104 for which minimally three data-points above detection threshold were available. Half-lives of patients 110, 111 and 112 with one or two data points above threshold were estimated as 0.7 days (K = 1).

Plasma viremia during one year of cART showed a two phase decline with an initial half-life (mean ± sem, days) of 1.6 ± 0.2 d and a second phase with a half-life of 8.1 ± 2.3 d and suppression of viremia predominantly below levels of 50 RNA copies/ml after 12 weeks of treatment (figure 4A). The fact that plasma viremia of patients 111 and 112 were slightly elevated at study week 48 was not considered a therapy failure, since plasma viremia returned to levels below 50 copies/ml at the next visit and remained suppressed during treatment for a further year (data not shown).

Whereas the total number of HIV-1 infected cells, as assessed by HIV-1 DNA levels, experienced comparably modest (74 ± 7%) and slow (t_1/2 _= 71 ± 60 d) decreases, in general more than 90% of HIV-1 RNA^+ ^cells decayed rapidly after therapy initiation. HIV-infected cell categories of elevated transcriptional activity (class II_High_, class-III_Extra_) became frequently undetectable after initiation of cART. However, their overall kinetics did not unequivocally match plain single phase exponential decay.

Similarly, decays of class-I_Low _and class-II_Medium _cells tended to flatten out during the course of ART. Therefore, half-lives were calculated assuming single phase decay kinetics to a plateau. As expected, class-III_Extra _cells showed rapid decays with an average half-life of 1.5 ± 0.6 d. Thus, the cell class matching criteria for strong ongoing productive infection declined with the fastest rate of all four cell classes. Half-lives of class II_High _cells varied from 1–13 days. Their average half-life (5.4 ± 2.6 d) was similar to that of class-I_Low _(3.2 ± 0.9 d) and class-II_Medium_: (4.1 ± 0.66 d). Hence, HIV-RNA^+ ^cells displaying basal viral transcription decayed more slowly than productively infected class-III_Extra _cells but with similar rates as class-II_High_.

Throughout the course of therapy, HIV-RNA^+ ^cells of class-II_High _and class-III_Extra _were largely outnumbered by cells of low viral activity (class-I_Low _plus class-II_Medium _>95% of HIV-RNA^+ ^cells) (figure 4G). Furthermore, contribution of class-II_High _to total cellular viral RNA burden (figure 4H) decreased from 44% at baseline to 30% during the initial month of therapy and declined to <10% by study week 24. Thus class-I_Low _and class-II_Medium _cells dominated the population of HIV-infected cells both in terms of cell-numbers as well as in regards to total cellular viral RNA burden.

## Discussion

In the present study highly sensitive, patient matched quantification of HIV-1 transcripts in total PBMC was combined with endpoint analysis at a single cell level. This enabled us to extend a previous analysis [22], in which we have observed that the vast majority of infected CD4-positive T-lymphocytes in peripheral blood in vivo expressed viral RNA at levels insufficient for virus production and could therefore be viewed as being of a latent phenotype. Conversely, virus production appeared to be confined to T-helper cells with HIV-mediated surface CD4-downregulation. Whereas the former analysis centered on differentiation between the different cell-types harboring viral RNA, the focus of the present work was, irrespective of cellular lineage, on quantitative aspects of HIV-transcription in vivo and its relation to viral decay kinetics during antiretroviral therapy. Applying a plain model and simple algorithms to analyze our complex dataset, we attained to assign HIV-RNA^+ ^cells to four transcriptional categories, namely productively infected cells carrying virions at their surface (class-III_Extra_), cells with elevated viral transcriptional activity fuelled by Tat/Rev- [20] and NEF-mediated [23] positive feedback (class-II_High_), cells expressing low levels of MsRNA and intermediate amounts of UsRNA (class-II_Medium_) and cells expressing solely UsRNA (class-I). The latter two classes match a strict definition of HIV-latency [15,22] since virion production may likely be precluded in these cells due to their low viral RNA content [15,22] and because these viral transcripts may potentially be confined to the nucleus [17].

Class-I_Low _viral RNA expression remained unchanged in a comparison of specimens from treated and untreated patients. Similarly, class-II_Medium _HIV-RNA content appeared constant throughout the course of therapy, whereas viral RNA expression of class-II_High _cells was significantly different in specimens before and during ART. This suggests that a highly active subgroup in this stratum was present in the absence of treatment which conceivably was identical or overlapping with the productively infected class-III_Extra_.

**Figure 5:**
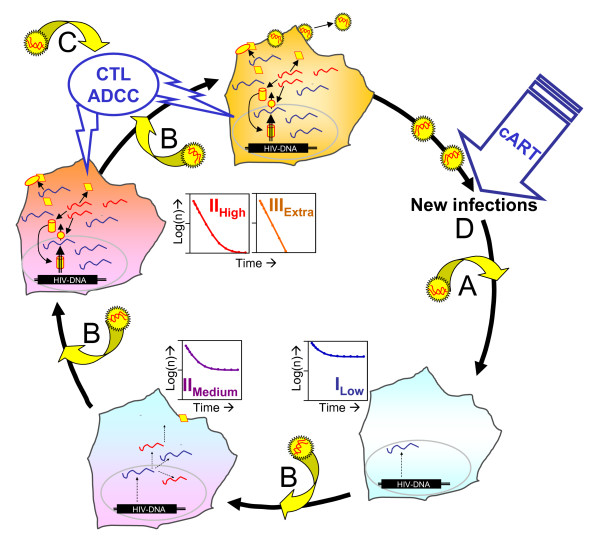
**A model of the turnover of HIV-RNA+ cells during the viral life-cycle**. The displayed cells symbolize HIV-1 infected cells of classes I_Low _(blue), II_Medium _(blue/magenta), II_High _(magenta/red) and III_Extra _(brown). Inserts within the circle symbolize the kinetics of each cell class during cART. Viral particles are depicted as black/yellow stars with the enclosed genomic RNA (vRNA-ex) in red. Viral proteins are shown as red/yellow diamonds, ellipses, circles and cylinders. Viral intracellular RNA is displayed as blue (UsRNA) or red (MsRNA) lines. Buckled arrows attached to viral particles signify the direct or indirect influences of activation due to ongoing viral replication and the presence of viremia on the viral life cycle [24,43-46]. A: Immune activation can promote post-entry events in resting CD4+ T cells harboring free viral cDNA such as integration and initiation of viral transcription [25,26,43]. B: Nonspecific immune activation can induce transcription in latently infected cells [27,47] and may result in a acceleration of viral replication [15,45]. C: CD8 T-cell mediated cytoxicity (CTL) [39,40] and humoral immunity [41], such as antibody-dependent cellular cytotoxicity (ADCC) and complement effector functions [28], are prone to attack HIV-1 infected cells expressing viral antigens. D: After initiation of cART, the viral life cycle is slowed primarily by preventing new infections but also by decreasing immune activation which results in decelerated rates of integration, detained induction of latency and reduced immune mediated HIV-specific cytotoxicity (A, B, C).

The stratification of viral RNA expressing PBMC as exercised in the present study provided only an approximate categorical model for the continuous patterns of HIV-1 transcription likely occurring in vivo. Furthermore, it is well possible that the cell classes characterized by different cell-frequencies and viral transcriptional activity may not reflect totally distinct and independent populations of infected cells. Rather they may represent cells 'caught' in different stages of the viral gene life cycle (figure 5, discussed further below) [24-28].

Notwithstanding the mathematical-model based nature of our analysis, and the considerable overlap observed in different classes of infected cells, we found good agreement of our results with independent estimates of HIV-1 RNA expression as employed by Haase and colleagues [29-33] who utilized sophisticated in situ hybridization to asses HIV-transcription *ex vivo *in lymphoid tissue. In particular they described two types of HIV-RNA^+ ^cells which persist during cART with viral RNA content of ≤ 5 copies [32,33] and cells expressing around 20 RNA copies [30]. These cell-types appear congruent to class-I_Low _cells and class-II_Medium _expressing on average 3.7 and 15 HIV-RNA copies/cell respectively. Furthermore, productively HIV-infected cells were estimated to contain on average 50–100 viral RNA-copies in lymphoid tissues whereas average levels of around 1000 RNA-copies were reported for virus producing PBMC cultures [29,32]. This is highly reminiscent of our observations that class-II_High _cells, which subsume at least two subcategories, displayed average expression levels of ~330 HIV-RNA copies/cell, which were found to be elevated up to 846 copies/cell at baseline.

All four HIV-RNA^+ ^cell categories identified in the current report showed rapid initial declines upon initiation of cART. For cells of high transcriptional activity, this was in concord with the notion that productively infected cells may be associated with the 1^st ^phase decay of plasma viremia [3]. Indeed, the decay of class-III_Extra _cells proceeded rapidly and their average half-live (1.5 ± 0.6 d) was virtually identical to the 1^st ^phase half-live of free plasma virus (1.6 ± 0.2 d). The half-lives of class-II_High _cells were slightly extended (5.4 ± 2.3 d). Unexpectedly kinetics of class-II_High _and, to some extent also of class-III_Extra _showed hyperbolic rather than plain negative exponential shapes. This finding may in part be explained by potential heterogeneities of cell classes as defined in our analysis. This applies in particular to class-II_High _cells which likely comprise both class-III_Extra _cells and a cell category of lower transcriptional activity escaping immunological clearance for longer time periods presumably due to moderate expression of viral antigens as predicted by mathematical models based on kinetics of plasma viremia [2,3].

Markedly contrary to initial expectations, class-I_Low _and class-II_Medium _kinetics proceeded similar to the course of class-II_High _cells. Whereas it was anticipated that they would show slow but steady exponential decline [2], we observed substantial initial decays with average half-lives of three to four days which were then followed by persistence. Rapid initial decay of latently infected cells had been previously observed in two reports by Blankson et al [34] and Strain et al [35] in which CD4^+ ^T-cells from acutely infected patients were examined by viral outgrowth assays. In consideration of the in vivo scarcity of provirus inducible by in vitro outgrowth assays [8], the cell types studied in these earlier reports may not be totally superposable to the two classes of latently infected cells identified in the present study. Hence, the half-lives calculated in the present analysis (0.7–6.3 days) were generally shorter than those reported by Blankson and colleagues (≥ 6 days). Nevertheless, our observation that two types of HIV-RNA^+ ^latently infected cells may shift from rapid to slower decay during the course of antiretroviral treatment appears to hold true also for cell subsets inducible by *in vitro *outgrowth assays. Such apparent variability in turnover of latently infected cells may be interpreted in two ways:

As discussed for class-II_High_, also cell-class-I_Low _and class-II_Medium _may be heterogeneous in terms of virological subcategories but also in reference to cellular lineage and/or in terms of their current state in the viral life cycle. Thus, class-I_Low _and class-II_Medium _may comprise cell-types with rapid turnover and long lived cells. Whereas the latter may be allocated to resting HIV-infected CD4^+ ^T-cells [36], the main candidates for weakly virus transcribing cells with a short half-life could be newly infected cells in a labile state of preintegration latency [9,37].

However, HIV-1 DNA levels, which have been reported to be dominated by nonintegrated molecules in PBMC of untreated patients [38], showed a much less pronounced decay than HIV-infected cells expressing viral RNA and no correlation with frequencies of class-I_Low _and class-II_Medium _cells (Spearman R = 0.26, p > 0.12), which were significantly correlated with each other (Spearman R = 0.67, p < 0.0001). This suggests that HIV-RNA^+ ^latently infected cells decayed with different kinetics than the majority of HIV-1 infected cells harboring nonintegrated viral DNA. Thus, the fast turnover of unintegrated HIV-DNA may not entirely account for the rapid initial decay of HIV-RNA^+ ^cells.

Therefore it is conceivable that turnover rates of HIV-1 infected cells may be intrinsically variable. In particular, they may be influenced by their cellular and humoral environment, which differs under conditions of cART and during viremia. Attenuation of antigenic stimulation due to cART results in declines of cellular [39,40] and humoral anti-HIV responses [41], which may decrease death rates of HIV-infected cells expressing viral antigens. Furthermore, generalized immune-activation by HIV-1 [42-46] may enhance viral replication itself by facilitating the transition from preintegration to genuine chromosomal proviral integration [37], or by activation of transcriptionally dormant provirus [47]. By accelerating the turnover of latently infected cells and driving the viral life cycle towards productivity (figure 5), feedback loops of various effects of HIV-1 on infected cells may potentiate viral replication [15,45]. In consequence of the decline of productively infected cells and of viremia, attenuation of antiviral immunity and systemic immune activation may contribute to the observed mitigation in turnover of latently infected cells.

## Conclusion

The present study was highly comprehensive in terms of PCR-targets and sample size despite limitations in patient number and in statistical resolution between different cell classes. Thus, it revealed important novel features of HIV-1 transcription and its relation with viral dynamics in vivo.

In summary, at least four categories of HIV-RNA^+ ^cells could be identified and assigned to distinct viral transcription patterns. Among these cell classes, latently infected cells represent the majority of HIV-1 infected PBMC both in the absence of treatment and during cART. This emphasizes the crucial role that latent HIV-1 reservoirs play not only in the maintenance of viral persistence during cART but, as precursors of productively infected cells, also under conditions of ongoing viral replication.

Foremost, we have gained evidence suggesting that at least two types of latently HIV-1 infected cells after initiation of cART decay with kinetics similar to those of productively infected cells. These rapid decays may be shaped by cellular and humoral immune responses against HIV and by HIV-induced activation which prevail before and early after initiation of cART but wither away when viral replication is stably suppressed. Verifying the hypothesis that decay of latently HIV-infected cells may not be constant but prone to influences from their cellular and humoral environment and identifying the factors governing the turnover of latently infected cells may help to devise novel therapeutic strategies to pursue the as yet elusive aim of eradicating HIV-1.

## Methods

### Patients and specimens

Five chronically HIV-1 infected therapy naïve patients initiating cART according to standard treatment guidelines (patient nr 103, 104, 110, 111, 112) were enrolled after informed consent was obtained. The study was approved by the ethics committee of the University Hospital Zürich. Patients were treated with zidovudine plus lamivudine (300 mg BID), and ritonavir boosted lopinavir (100/400 mg BID), except for patient 103 who replaced ritonavir/lopinavir by efavirenz (daily 1 × 600 mg) at week 32. Median (range) baseline clinical parameters were 147(116; 262) CD4 T-lymphocytes/μl blood and 158'000 (14'300; 203'500) viral RNA copies/ml plasma. PBMC were purified as described [15] and aliquots (n = 8–14) were distributed into equal proportions ranging from 1–4 million cells/tube. Further serial five-fold dilutions of PBMC (12–24 replicates each; 5×, 25×, 125×, 625×, 3125× dilutions) were prepared and diluted PBMC were supplemented with uninfected CEM cells [48] (3 × 10^5 ^cells/aliquot) to avert cell loss in dilutions. Cells were stored at -80°C as dry pellets.

### Nucleic acid analysis

RNA was extracted (RNeasy, Qiagen Hombrechtikon, CH) using two protocols in parallel [12,15]. Total PBMC RNA was extracted by the standard procedure [49] and specific isolation of PBMC-associated extracellular, virion-enclosed HIV-RNA (vRNA-ex) was performed by nucleolytic digestion of cellular nucleic acids while RNA enclosed in viral envelopes remain protected [12,15,18]. RNA was eluted in a volume of 0.1 ml.

**Figure 6:**
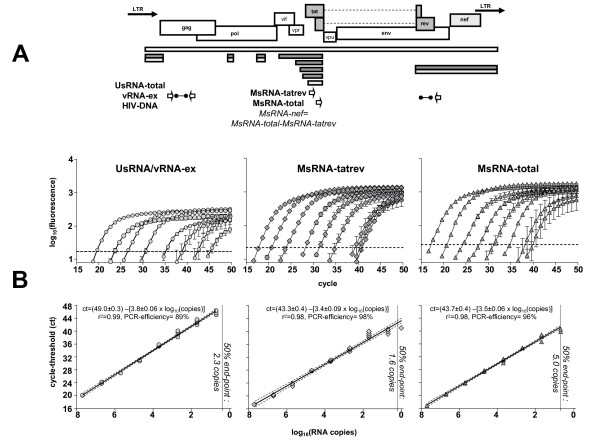
**PCR-Quantification of HIV-RNAs**. A: Location of primers and probes used for PCR. Labeled bars depict viral genes and the unlabelled bars below show exons of viral transcripts quantified as previously described [15]. White: genomic RNA spanning the entire transcriptional unit, grey: multiply spliced RNAs encoding tat and rev (dark grey) or nef (light grey). Arrows show the location of PCR primers and barbells the positioning of fluorescent probes. Note that MsRNA-nef was calculated as the difference of copy number for MsRNA-total and MsRNA-tatrev as described previously [15] (formula indicated in italic letters). B: Performance of PCR assays. Patient-specific *in vitro *transcribed UsRNA and MsRNA prepared from templates isolated from patient 104 and quantified photometrically was subjected to RT-PCR using patient specific primers as listed in table 3. Ten-fold dilutions ranging from 5 × 10^7 ^– 5 copies were run in replicates of two (5 × 10^7 ^– 5 × 10^4 ^copies) or four (5000–5 copies). Upper panels show amplification plots (mean ± standard error) with the amplification threshold (broken lines) as calculated by default settings of the software used (i-cycler, Biorad). In the lower panels log-transformed copy numbers were plotted against cycle-threshold (ct) values of each replicate. Linear regression analysis as shown by the resulting equations for black lines (broken lines depict 95% confidence intervals) revealed high correlation coefficients of standard curves. PCR efficiencies as deduced from slopes ranged from 89%–96%. Sensitivities of PCR assays (grey vertical lines, italic numbers) were estimated by determination of 50% endpoints [51]. All assays approached single copy sensitivities as previously documented [15,22] with assays for MsRNA-tatrev (1.6 copies) being the most sensitive ones followed by PCR for UsRNA/vRNA-ex (2.3 copies) and the assay for MsRNA-total (5 copies). PCR assays for the other patients performed similarly (data not shown).

PCR primers and fluorescent probes mapping to the *gag *gene were used to quantify UsRNA, vRNA-ex and HIV-DNA [50]. PCR strategies to quantify viral nucleic acids are shown in figure 6A. Analysis of MsRNA was performed [15] utilizing a PCR for MsRNA encoding Nef, Tat and Rev (MsRNA-total) and an assay for MsRNA encoding Tat and Rev (MsRNA-tatrev). Subtracting copy numbers of MsRNA-total from MsRNA-tatrev allowed for calculation of MsRNA encoding Nef (MsRNA-nef) [15]. Some specimens with very low HIV-RNA content, showed MsRNA-total copy numbers smaller or equal to MsRNA-tatrev, in this case MsRNA-nef was assumed being undetectable. Since values for MsRNA-tatrev and MsRNA-nef showed similar kinetics, data analysis of the sum of both parameters, referred to as MsRNA, was performed. HIV-1 PCR primers used were individually adjusted to their binding sites for each patient's predominant quasispecies as described [22] (table 3). Real time RT PCR assays were performed using a protocol which approached single copy sensitivity as previously reported [15,18,22] and as exemplified in figure 6B. HIV-DNA was amplified as described [22]. PCR was performed in duplicate reactions using 10–20 μl RNA per specimen. PCR data were interpolated from patient-matched RNA standard-curves [22]. HIV-RNA in plasma was measured using Amplicor HIV-Monitor version 1.5 (Roche Diagnostics, Basel, Switzerland).

**Table 3:** Patient matched PCR primers

		Position ^C^			
Primer-ID^a^	Function^b^	5'	3'	Sequence ^D^	Analysis of patient no^E^
Ts5'gag	Sense-primer UsRNA/vRNA-ex	1372	1397	**CAAGCAGCCATGCAAATGTTAAAAGA**	103, 104, 110^5^, 111
				CAAGCAGCTATGCAAATGTTAAAAGA	112

Boe3tq	Antisense-probe UsRNA/vRNA-ex	1430	1405	**f-CTATCCCATTCTGCAGCTTCCTCATT-q**	104, 110^E^, 111, 112
				f-TCTATCCCATTCTGCAGCTTCTTCATT-q	103

Boe2	Antisense-primer UsRNA/vRNA-ex	1488	1467	**TCCCCTTGGTTCTCTCATCTGG**	104, 110^E^, 111, 112
				TCCCCTTGGTTCCCTTATCTGG	103

Skcc1b	cDNA-primer, UsRNA/vRNA-ex	1513	1486	**TACTAGTAGTTCCTGCTATGTCACTTCC**	103, 110^E^, 111, 112
				TCCCCTTGGTTCCCTTATCTGG	104

Mf1	Sense-primer MsRNA-tatrev	5956	5979	**CTTAGGCATCTCCTATGGCAGGAA**	103, 104, 111, 112
				ATTAGGCATCTCCTATGGCAGGAA	110^E^

Ts5'allspl	Sense-primer MsRNA-total	5978	6001	**AAGAAGCGGAGACAGCGACGAAGA**	103, 104

Mf84	Sense-primer MsRNA-total	6012	6045	**ACAGTCAGACTCATCAAGTTTCTCTATCAAAGCA**	111
				ACAGTAAGACTCaTCAAGCTTCTCTATCAAAGCA	110^E^
				CAGTCAGACTCATCAAG**C**TTCTCTATCAAAGCA	112

mf2tq	Antisense-probe MsRNA-total MsRNA-tatrev	8421	8399	f-**TTCCTTCGGGCCTGTCGGGTCCC**-q	104
				f-TTCCTTCGGGCCTGTCTGGTCCC-q	103

Mf226tq	Sense-Probe MsRNA-total MsRNA-tatrev	8397	8414	f-**AGGGGACCCGACAGGCCC**-q	110^E^, 112
				f-AGGGGA*CGA*CCCGACAGG-q	111

Mf83	Antisense/cDNA-primer MsRNA-total MsRNA-tatrev	8459	8433	**GGATCTGTCTCTGTCTCTCTCTCCACC**	111
				TGATCTGCCTCTGTCTTGCTCTCCACC	103
				GGATGTGTCTCTGTCTCTGTCTCCACC	104
				TGATGTGTCTCTGTCTCTCTCTCCACC	110^E^
				GGATCTGTCTCTGTCTCTGTCCCCACC	112

^A ^ID denominates identification of primers according to internal nomenclature in the authors laboratory[22].

^B ^Oligonucleotide function; Sense/antisense-primer: oligonucleotide of the respective polarity used for amplification, sense/antisense probe: fluorogenic probe of the respective polarity used for detection of genuine amplification products, cDNA-primer: oligonucleotide of negative polarity used for reverse transcription prior to amplification. In assays for UsRNA/vRNA-ex separate cDNA and antisense primers were used except for patient 112 (skkcc1b was used for cDNA synthesis and PCR). In assays for MsRNAs, the same primer was used for reverse transcription and amplification.

^C ^Numbering on the Hxb2 genome according to the software provided by the Los Alamos HIV database; (SEQUENCE LOCATOR, ).

^D ^Sequences are indicated 5' to 3' from left to right. Underlined positions show deviation from the standard sequence (HXB2 or clade B consensus) (indicated in bold letters). f = Fluorescein-moiety attached to the DNA-probe, q= Fluorescence quenching moiety attached to the DNA-probe.

^E ^Note that PCR primers of patient 110 have been previously reported [22]. In this earlier study, in which patient 110 has been referred as *"patient 08"*, different time points and specimens have been analyzed with a focus on the cellular origin of HIV-1 transcription.

### Calculations and statistics

Statistics were performed using GraphPad Prism4.0 software (GraphPad Software, San Diego, CA).

RNA copy-numbers were calculated as the mean of duplicate PCR measurements, extrapolated to copy numbers per tube (5–10-fold multiplication) and normalized to the input of PBMC. In HIV-RNA^+ ^specimens near the detection limit of the test, quantification of HIV-RNA was censored to avoid gross overestimation: If only one duplicate PCR was positive and/or nominal copy numbers were below 1 copy per reaction, it was assumed that 1 RNA copy had been present per tube.

Stratification and assignment of specimens to different transcriptional classes were executed according to criteria compiled in table 1. Frequencies of HIV-RNA^+ ^cells were calculated by 50% endpoint analysis [22] according to the method described by Muench and Reed [51]. Specific RNA contents in different transcriptional classes were calculated by a stepwise "bottom up" approach in which the contribution of the different classes of HIV-RNA expressing cells was dissected as described in figure 2: Since cells of frequent transcriptional classes co-occurred with cells of rarer classes, the contribution of the first had to be subtracted from the total copy numbers to calculate the copy numbers of the latter. After this subtraction HIV-RNA copy numbers in a given specimen were normalized to its content of HIV-RNA^+ ^cells. Rarely this resulted in nominally negative values which were censored to 1 copy per cell, to allow further analyses using logarithmic transformation.

Statistical comparisons between groups were performed using nonparametric tests; the Mann-Whitney test for unpaired data-sets, Wilcoxon signed rank tests for paired analyses and, when adjustment for multiple testing was applied, one way Anova and a Friedman post test analyzing all possible combinations was applied.

## Abbreviations

cART: combination antiretroviral therapy; PBMC: peripheral blood mononuclear cells; UsRNA: unspliced HIV-RNA; MsRNA: multiply spliced HIV-RNA; MsRNA-total: MsRNA encoding tat, rev and nef; MsRA-tatrev: MsRNA encoding Tat and Rev; MsRNA-nef: MsRNA encoding Nef; vRNA-ex: virus encapsidated cell-associated unspliced HIV-RNA; I_Low_, II_Medium_, II_High_: HIV-infected cells with low, intermediate and expression levels of HIV-RNA respectively; sem: standard error of the mean; d: days.

## Competing interests

The authors declare that they have no competing interests.

## Authors' contributions

MF designed and developed the algorithm for single cell transcriptional analysis, carried out the data analysis, conceived the study and wrote the manuscript. BJ participated in sequence analysis, design of PCR primers and drafting of the manuscript. BN participated in performing the laboratory work for PCR and sequence analysis. PK participated in data analysis, drafting the manuscript and development of single cell transcriptional analysis. RH participated in patient-recruitment and -care. VvW participated in statistical analysis and drafting the manuscript. MA participated in statistical analysis. RW participated in drafting the manuscript and designing the present study. HFG participated in conceiving the study, and participated in its design and coordination and helped to draft and finalize the manuscript. All authors read and approved the final manuscript.
